# Retrospective investigation of tacrolimus combined with an anti‐tumor necrosis factorα antibody as remission induction therapy for refractory ulcerative colitis: Efficacy, safety, and relapse rate

**DOI:** 10.1002/jgh3.12197

**Published:** 2019-07-05

**Authors:** Ayumi Ito, Teppei Omori, Shinichi Nakamura, Katsutoshi Tokushige

**Affiliations:** ^1^ Department of Gastroenterology Tokyo Women's Medical University Tokyo Japan

**Keywords:** refractory ulcerative colitis, remission induction therapy, combined therapy with tacrolimus and an anti‐TNFα antibody

## Abstract

**Background and Aim:**

Combined therapy with tacrolimus (TAC) and an anti‐tumor necrosis factorα (TNFα) antibody is used to induce remission in ulcerative colitis (UC) patients who have not responded to monotherapy with either drug. We evaluated the efficacy and safety of combined therapy, as well as the relapse rate.

**Methods:**

Combined therapy was performed to induce remission in UC patients showing an inadequate response to monotherapy with TAC or an anti‐TNFα antibody. The following items were assessed retrospectively: (i) clinical characteristics, (ii) the remission induction rate, (iii) the relapse rate, and (iv) adverse events.

**Results:**

Combined therapy induced remission in 7 of the 12 patients (58.3%). There were no significant differences in clinical characteristics between the patients with and without the successful induction of remission. However, the number of female patients tended to be higher in the remission group than in the nonremission group. The remission group also showed trends of a lower clinical activity index (Lichtiger index; CAI) on admission and before combined therapy and a lower total dose of prednisolone during hospitalization. The 1‐year relapse rate was 33.3%. Adverse events due to combined therapy included renal impairment (*n* = 2), tremors (*n* = 2), influenza (*n* = 1), and a positive cytomegalovirus antibody test (*n* = 3). None of these events were serious.

**Conclusions:**

Combined therapy was effective in more than half of the patients with refractory UC who had not responded to monotherapy. Our findings suggest that combination therapy may be a new, third option for the treatment of refractory UC.

## Graphical abstract

Combined therapy with tacrolimus and an anti‐TNFa antibody is used to induce remission in ulcerative colitis patients who have not responded to monotherapy with either drug. We evaluated the efficacy and safety of this combination therapy, as well as the relapse rate.

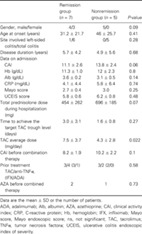

## Introduction

Treatment with tacrolimus (TAC) or anti‐tumor necrosis factorα (TNFα) antibodies is effective as a remission induction therapy in patients with prednisolone (PSL)‐dependent or PSL‐resistant refractory ulcerative colitis (UC).^1^ In UC patients receiving TAC therapy, the short‐term remission rate was reported to be 52.6%, and the postrelapse colectomy rate was 69% at 1 year after the induction of remission.^2^, ^3^ In UC patients receiving infliximab (IFX), an anti‐TNFα antibody, the short‐term remission rate was 69%, and remission was maintained in 45% of patients at 54 weeks after induction.^4^ In UC patients treated with adalimumab (ADA), another anti‐TNFα biologic, the remission rate was 49.1%, and remission was maintained in 69.8% of patients at 52 weeks after induction.^5^

These results indicate that, in 40–50% of UC cases, remission is not achieved by monotherapy with TAC or an anti‐TNFα antibody, and the longer‐term outcome is not good. Some patients who do not respond to monotherapy with TAC or an anti‐TNFα antibody are switched to the other drugs (i.e. switched from TAC to an anti‐TNFα antibody or vice versa).^1^ Unfortunately, the results of switching therapy are often unsatisfactory, and surgical treatment may be required. In patients with severe UC that has not responded to medical treatment, the risk of complications such as toxic megacolon and the influence on the patient's overall condition need to be taken into consideration, often resulting in the selection of surgery.^1^, ^2^

However, surgery for UC is associated with many complications.^6^, ^7^ For example, the increase of stool frequency after surgery can have a marked influence on the quality of life (QOL) of patients.^8^, ^9^ Therefore, it is crucial to identify the best drug therapy to avoid those risks. At our hospital, combined therapy with TAC and an anti‐TNFα antibody has been utilized in patients after hospitalization to induce remission when it is not achieved by monotherapy with either drug. In the present study, we retrospectively evaluated the efficacy and safety of combined therapy with TAC and an anti‐TNFα antibody, as well as the relapse rate after such a therapy.

## Methods

In the 12 patients who received the combined therapy with TAC and an anti‐TNFα antibody (TAC + anti‐TNFα therapy) at our hospital from April 2016 to March 2018, we evaluated the following points: (i) the overall remission induction rate, (ii) differences of clinical characteristics between the groups with and without induction of remission, (iii) the relapse rate after remission, and (iv) the incidence of adverse events. In this study, the patients receiving combined therapy initially underwent monotherapy with TAC or an anti‐TNFα antibody; when monotherapy was found to be ineffective, the other drug was added concomitantly with continuation of the monotherapy drug. In patients receiving TAC monotherapy, concomitant administration of an anti‐TNFα antibody was initiated if the response was insufficient approximately 1 week after achieving a high trough blood level of TAC (≥10 ng/dL). In patients receiving anti‐TNFα monotherapy, concomitant administration of TAC was started if the response to anti‐TNFα antibody was insufficient after the second dose. During combined therapy, a high trough blood level of TAC (≥10 ng/dL) was maintained. Symptoms were evaluated using the Lichtiger index (CAI).^10^ Patients with an insufficient response to monotherapy were defined as those in whom the CAI was improved by ≤3 points after starting monotherapy.

**Figure 1:**
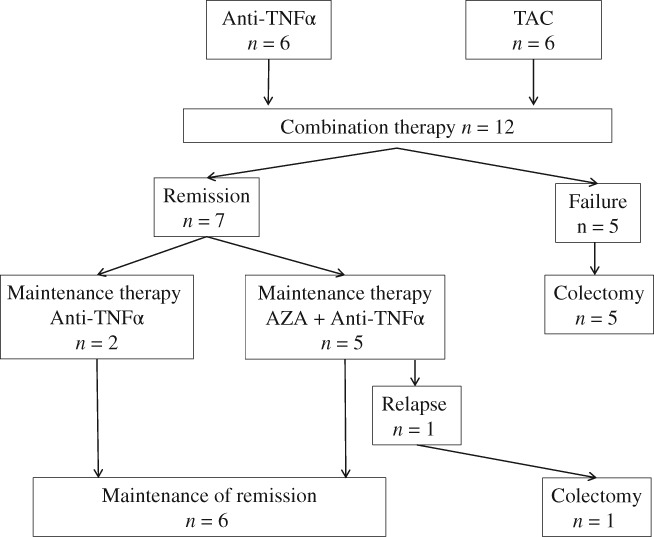
Patient flow. Twelve UC patients received the combination therapy. Seven patients achieved remission. Finally, six patients were maintained on remission state. AZA, azathioprine; TAC, tacrolimus; UC, ulcerative colitis.

Remission was defined as CAI ≤4 at 4 weeks or more after the start of remission induction with TAC + anti‐TNFα therapy. Relapse was defined as follows: the need for high‐dose PSL, switching of anti‐TNFα agents, readministration of TAC or readministration of TAC at a higher dose for remission induction, or performance of surgery. The Mayo score and ulcerative colitis endoscopic index of severity (UCEIS) score were determined as colonoscopic scores.^11^, ^12^ In the patients who achieved remission, TAC was stopped after mucosal healing, and maintenance therapy was performed with anti‐TNFα monotherapy or the combination of an anti‐TNFα antibody and azathioprine (Fig. 1). Mucosal healing was defined as a Mayo score of 0–1.^13^

### *Statistical analysis*

Results are expressed as the mean ± SD or the number of patients. The Wilcoxon test was used for comparisons between the two groups, and differences were considered to be significant at *P* < 0.05. JMP Pro12 (Statistical Discover, SAS) was used as the statistical program. The cumulative relapse rate of remission cases was calculated using the Kaplan–Meier (K‐M) method.

### *Ethical considerations*

The protocol of this study was reviewed and approved by the Ethics Review Committee of Tokyo Women's Medical University. Informed consent was obtained from all patients.

## Results

### *Remission induction rate and clinical characteristics*

The characteristics of all patients are summarized in Table 1. The average age (mean ± SD) was 37.4 ± 23.6 years; nine patients (75%) were male, and the mean duration of UC was 5.3 ± 4.6 years. On admission, the mean CAI was 12.2 ± 2.8. With regard to colonoscopic scores, the Mayo score was 2.8 ± 0.3, and the UCEIS score was 6.0 ± 0.7 on admission. CAI was 9.0 ± 2.2 at the time of starting combined therapy. Before receiving combined therapy, six patients (50%) each had been on TAC monotherapy and anti‐TNFα monotherapy. The anti‐TNFα antibody was IFX in five patients (83.3%) and ADA in one patient (16.7%).

**Table 1:** Clinical characteristics of total UC patients

Patient	Effect	Gender	Age at onset (years)	Site involved left‐sided/total colitis	Disease duration (years)	Admission CAI	Admission Hb (g/dL)	Admission Alb (g/dL)	Admission CRP (mg/dL)	Admission Mayo score	Admission UCEIS
①	Remission	Male	18	Total colitis	11	16	11.1	3.4	11.47	3	7
②	Remission	Female	18	Total colitis	11	8	12.1	4.0	1.33	3	6
③	Remission	Male	27	Total colitis	6	11	13	3.5	0.33	2	5
④	Remission	Male	15	Left sided	3	12	10.6	4.0	9.18	3	6
⑤	Remission	Female	19	Total colitis	2	9	9.9	3.8	2.65	2	5
⑥	Remission	Female	73	Total colitis	0.1	10	11.9	3.7	3.8	3	6
⑦	Remission	Male	49	Total colitis	7	12	10.5	3.4	0.32	3	6
⑧	Non‐remission	Male	70	Total colitis	0.1	18	9.9	2.8	9.8	3	6
⑨	Non‐remission	Male	63	Total colitis	0.5	12	9.6	2.7	1.12	3	6
⑩	Non‐remission	Male	61	Total colitis	2	14	14	2.7	15.21	3	7
⑪	Non‐remission	Male	17	Total colitis	10	13	11.9	3.7	2.34	3	5
⑫	Non‐remission	Male	19	Total colitis	12	12	14.7	3.8	0.67	3	7

Patient	Total prednisolone dose during hospitalization (mg)	Time to achieve the target TAC trough level (days)	TAC average dose (mg/day)	CAI before combination therapy	Before combination therapy	AZA before combination therapy
①	535	2	5.08	12	TAC	+
②	0	10	10.64	6	IFX	−
③	365	3	14.3	8	ADA	+
④	885	2	6.32	9	IFX	−
⑤	420	2	7.51	7	TAC	−
⑥	525	2	4.65	7	TAC	−
⑦	450	1	4.05	9	IFX	−
⑧	595	1	2.04	14	TAC	−
⑨	580	2	4.52	9	IFX	−
⑩	840	3	0.88	10	TAC	−
⑪	520	1	6.6	10	IFX	−
⑫	945	1	7.51	8	TAC	+

ADA; adalimumab; Alb, albumin; AZA, azathioprine; CAI, clinical activity index; CRP, C‐reactive protein; Hb, hemoglobin; IFX; infleximab; Mayo, endoscopic activity index; TAC, tacrolimus; UC, ulcerative colitis; UCEIS, ulcerative colitis endoscopic index of severity.

TAC + anti‐TNFα therapy achieved remission in 7 of 12 patients, resulting in a remission induction rate of 58.3%. The following differences were noted between the remission group and the nonremission group (Table 2): female patients (four men and three women in the remission group *vs* five men and zero women in the nonremission group; *P* = 0.09), a lower CAI on admission (11.1 ± 2.6 in the remission group *vs* 13.8 ± 2.4 in the nonremission group; *P* = 0.06), a lower CAI before combined therapy (8.2 ± 1.9 in the remission group *vs* 10.2 ± 2.2 in the nonremission group; *P* = 0.1), and a lower total dose of PSL (mg) during hospitalization (454 ± 262 in the remission group *vs* 696 ± 185 in the nonremission group; *P* = 0.07).

**Table 2:** Comparison between remission group and nonremission group

	Remission group (*n* = 7)	Nonremission group (*n* = 5)	*P*‐value
Gender, male/female	4/3	5/0	0.09
Age at onset (years)	31.2 ± 21.7	46 ± 25.7	0.41
Site involved left‐sided colitis/total colitis	1/6	0/5	0.28
Disease duration (years)	5.7 ± 4.2	4.9 ± 5.6	0.68
Data on admission			
CAI	11.1 ± 2.6	13.8 ± 2.4	0.06
Hb (g/dL)	11.3 ± 1.0	12 ± 2.3	0.8
Alb (g/dL)	3.6 ± 0.2	3.1 ± 0.5	0.14
CRP (mg/dL)	4.1 ± 4.4	5.8 ± 6.4	0.74
Mayo score	2.7 ± 0.4	3.0	0.25
UCEIS score	5.8 ± 0.6	6.2 ± 0.8	0.48
Total prednisolone dose during hospitalization (mg)	454 ± 262	696 ± 185	0.07
Time to achieve the target TAC trough level (days)	3.0 ± 3.1	1.6 ± 0.8	0.27
TAC average dose (mg/day)	7.5 ± 3.7	4.3 ± 2.8	0.022
CAI before combination therapy	8.2 ± 1.9	10.2 ± 2.2	0.1
Prior treatment TAC/anti‐TNFα, (IFX/ADA)	3/4 (3/1)	3/2 (2/0)	0.58
AZA before combined therapy	2	1	0.73

Data are the mean ± SD or the number of patients.

ADA, adalimumab; Alb, albumin; AZA, azathioprine; CAI, clinical activity index; CRP, C‐reactive protein; Hb, hemoglobin; IFX, infliximab; Mayo score, Mayo endoscopic score; ns, not significant; TAC, tacrolimus; TNFα, tumor necrosis factorα; UCEIS, ulcerative colitis endoscopic index of severity.

### *Relapse rate*

**Figure 2:**
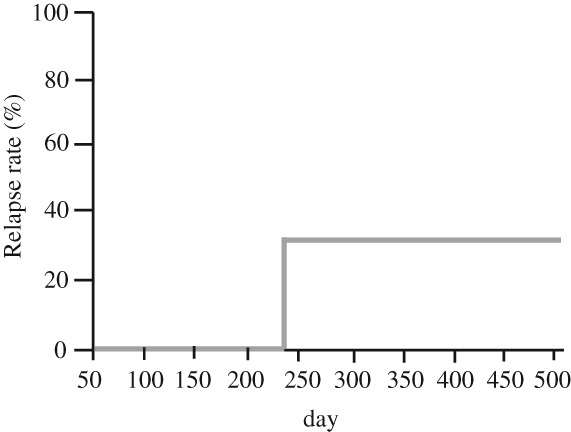
Relapse rate. One‐year relapse rate was 33.3% in seven remission cases.

In the seven patients who achieved remission, the relapse rate was evaluated over a mean observation period of 277 ± 171 days. Relapse occurred in only one patient at 234 days after the induction of remission. Analysis using the K‐M method showed that the 1‐year relapse rate was 33.3% (Fig. 2). The relapsed patient subsequently underwent surgery.

### *Adverse events*

Adverse events were observed in 8 of 12 patients (66%), including renal impairment (*n* = 2), tremors (*n* = 2), influenza A (*n* = 1), and a positive cytomegalovirus antibody test (*n* = 3) (Table 3). In the patient who developed influenza A, TAC + anti‐TNFα therapy was discontinued, leading to a discontinuation rate of 8.3% (*n* = 1). In the patients with renal impairment, tremors, and a positive cytomegalovirus antibody test, the dose of TAC or PSL was adjusted, and combined therapy was continued.

**Table 3:** Adverse events

Adverse events	Number (%)
Total	8 (66.6)
Renal impairment	2 (16.6)
Tremor	2 (16.6)
Influenza A	1 (8.3)
Cytomegalovirus positivity	3 (25)

## Discussion

### *Remission induction rate and clinical characteristics*

TAC is an immunosuppressant that mainly affects activated T cells.^14^ TNFs are inflammatory cytokines mainly released by activated macrophages,^2^ and anti‐TNF antibodies not only inhibit TNFs as neutralizing antibodies but also eliminate the activated macrophages. Thus, the drugs used in TAC + anti‐TNFα therapy have different mechanisms of action, and the combination is expected to have an additive effect. In this study, combined therapy was performed in 12 patients, of whom 6 patients each had already received TAC monotherapy or anti‐TNFα monotherapy.

Combination therapy was performed after judging that the effect of monotherapy was insufficient in cases based on a CAI score improvement of less than three points. The adoption of combination therapy is required in serious cases of UC, in which remission is not achieved by monotherapy alone.

The remission induction rate achieved with TAC + anti‐TNFα therapy was 58.3%. All patients in whom remission was not induced by combined therapy subsequently underwent surgery (Fig. 1). It is difficult to directly compare the present remission induction rate with the rates in published reports on patients receiving TAC or anti‐TNFα monotherapy because different evaluation methods were used in our study and previous studies. However, the remission induction rate was 52.6% at Week 10 in patients receiving TAC monotherapy, 69% at Week 8 in patients receiving IFX (an anti‐TNFα antibody) monotherapy, and 49.1% at Week 8 in patients receiving ADA (an anti‐TNFα antibody) monotherapy (Table 4).^2^, ^4^, ^5^ The remission induction rate for patients receiving TAC + anti‐TNFα therapy in our study was slightly higher than that reported for patients receiving TAC monotherapy but slightly lower than that reported for patients receiving IFX monotherapy. A reason for this result may be the differences of patient characteristics between the present study and the previous studies of IFX monotherapy (ACT1 and 2). As most of the patients in the ACT1 and 2 studies had moderate disease rather than severe disease, it could be expected that the remission induction rate would be higher in those studies. In addition, the definition of remission was different, and remission could be achieved more easily in the ACT1 and 2 studies. We think that these points might have led to differences in the remission induction rates between our study and previous studies.^4^ In the patients receiving combined therapy in our study, clinical disease activity was similar or more severe than in the patients receiving TAC or anti‐TNFα monotherapy in previous studies (Table 4).

**Table 4:** The results of TAC, anti‐TNF, and TAC and anti‐TNF combination therapy

Author	Therapy	Sample size	Disease activity	Concomitant medications for UC	Definition of clinical remission	Remission rate (%)	Relapse rate (surgical rate)
Ogata *et al*.^2^	Tacrolimus (high trough group)	*n* = 42	Moderately or severe active UC	5‐ASA, PSL	Clinical remission was defined as a DAI score ≤2, with no individual subscore >1	52.6	69% (surgical rate)
Rutgeerts *et al*.^4^	IFX	*n* = 364	Moderately or severe active UC (Mayo score 24 of 6–12 points)	5‐ASA, PSL, immunosuppressants (AZA, mercaptopurine)	Clinical remission was defined as a total Mayo score of 2 points or lower, with no individual subscore exceeding 1 point	69	45%
Muñoz‐Villafranca *et al*.^5^	ADA	*n* = 53	Moderately or severe active UC (Mayo score/(mean, SD) 8.92/1.47; partial Mayo score 6.6/1.13	5‐ASA, steroid, immunosuppressants	Clinical remission was defined as a partial Mayo score ≤ 2 plus blood‐in‐the‐stool assessment at value 0	49.1	60.3%
Herrlinger *et al*.^16^	Change from TAC to IFX	*n* = 19	Severe active UC (CAI > 10)	immunosuppressants (AZA, methotrexate)	Clinical remission was defined as a CAI ≤L3 points	25	58% (surgical rate)
Maser *et al*.^15^	Change from IFX to CyS/CyS to IFX	*n* = 19	Severe active UC (CAI > 10)	PSL, immunosuppressants	Remission was strictly defined as a normal number of bowel movements, the absence of rectal bleeding, and a discontinuation of corticosteroids within 3 months and a Lichtiger score of 3 or less	40/40.7	NA
Our study	Combination of tacrolimus and anti‐TNFα	*n* = 12	Severe active UC CAI 12.2 ± 2.8	5‐ASA, steroid, AZA	Clinical remission was defined as CAI less than or equal to 4	58.3	33.3%

5‐ASA, 5‐aminosalicylic acid; ADA, adalimumab; AZA, azathioprine; CAI, Lichtiger score; CyS, cyclosporine; DAI, disease activity index score; IFX, infliximab; Mayo score, Mayo endoscopic score; NA, not available; PSL, prednisolone; TAC, tacrolimus; TNFα, tumor necrosis factorα; UC, ulcerative colitis.

The patients we investigated all had refractory UC. Despite this, TAC + anti‐TNFα therapy achieved a remission induction rate similar to the rates for patients with less severe disease receiving TAC or anti‐TNFα monotherapy in previous studies. This suggests that combined therapy may be a viable treatment option. In patients with moderate or severe UC, PSL should be administered first.^1^ In patients with PSL‐resistant or PSL‐dependent refractory UC, a calcineurin inhibitor (TAC or cyclosporine) or an anti‐TNFα drug should be selected as second‐line therapy. If the response to second‐line therapy is insufficient, further medical treatment or surgery should be considered. However, the outcome of third‐line medical therapy is often poor.^1^, ^15^, ^16^ Conventionally, the drug that was not used for second‐line therapy is tried for third‐line therapy. According to published reports, when remission is not induced by anti‐TNFα therapy, and treatment is changed to cyclosporine, the remission induction rate with TAC therapy is only 40%.^15^ Likewise, if remission is not induced by TAC, and treatment is changed to anti‐TNFα therapy, the remission induction rate with anti‐TNFα therapy is only 40.7%.^16^

If the response to second‐line therapy is inadequate, surgery is often selected after taking into consideration its potential influence on the patient's general condition and the likely postoperative course. Some studies have demonstrated the efficacy of third‐line medical therapy, but the ECCO guidelines state that further evaluation should be performed in specialized institutions.^1^, ^15^, ^17^

In the present study, TAC + anti‐TNFα therapy was evaluated as a third‐line therapy for patients with refractory UC. There was a difference in the third‐line therapy between our study and previous studies. Instead of TAC or anti‐TNFα monotherapy, as was carried out in the past, TAC was combined with an anti‐TNFα agent to achieve induction of remission in our study. This combination is effective one patient of third‐line therapy, the remission induction rate was 58% in patients receiving TAC + anti‐TNFα therapy, suggesting that this combination is effective one patient of third‐line therapy.

Regarding the combination of TAC and an anti‐TNFα antibody, there has only been one published case report about a UC patient who received this combination therapy.^18^

In contrast, there have been several reports about disease control using the combination of TAC and an anti‐TNFα antibody in patients with rheumatoid arthritis.^19^, ^20^ One study evaluated the efficacy of this combination in 624 patients with rheumatoid arthritis who had shown an inadequate response to anti‐TNFα monotherapy.^19^ Remission was induced or disease activity was reduced in 391 patients (62.7%) at 24 weeks after the start of combined therapy, and this combination was shown to be effective. Obviously, the effect of this treatment in patients with rheumatoid arthritis cannot be translated directly to UC, but the data are suggestive.

The number of patients in the present study was small. While comparison of patient characteristics between the remission group and the nonremission group demonstrated no significant differences, female patients tended to be more frequent in the remission induction group than in the nonremission group, and the remission‐induction group also showed trends of a lower CAI on admission, lower CAI before combined therapy, and lower total dose of PSL compared with the nonremission group. Previous studies have found no significant difference in the severity or course of UC between men and women.^21^ In the present study, the proportion of female patients achieving remission was higher, but this may well have been because of the small patient population. Our finding regarding CAI before combined therapy and total dose of PSL suggested that combined therapy may not be effective when UC is more severe. In the future, data on more patients should be collected from multiple institutions, and further evaluation of the characteristics associated with the response to TAC + anti‐TNFα therapy should be performed.

### *Relapse*

The relapse rate was evaluated in the seven patients in whom remission was induced by combination therapy. The mean observation period was 277 ± 171 days, and relapse occurred in one patient at 234 days after induction of remission. The 1‐year relapse rate was 33.3% using the K‐M method (Fig. 2). The patient who relapsed subsequently underwent surgery for UC. It was reported that the colectomy rate due to relapse was 69% at 1 year after remission in UC patients treated with TAC therapy.^3^ In a study of anti‐TNFα therapy, the remission maintenance rate was 45% at 54 weeks after induction of remission by IFX.^4^ It was also reported that the remission maintenance rate was 69.8% at 52 weeks after induction of remission by ADA (Table 4).^5^ Because the observation period was short, direct comparison of results between our study and other studies was difficult.

However, given the reported relapse rates for TAC monotherapy and anti‐TNFα monotherapy, the relapse rate of 33.3% in combined therapy suggests that this might be effective. Because our result was obtained over the short term in a small number of patients, further evaluation of relapse after remission induction with TAC + anti‐TNFα therapy should be performed in a larger patient population over a longer period.

### *Adverse events*

No serious adverse events occurred in this study. In one patient who developed influenza A, treatment with TAC and the anti‐TNFα antibody was discontinued, and surgery was performed for UC instead. In two patients with renal impairment and two patients with tremors, the adverse events were improved by reducing the dose of TAC. In three patients who became positive for cytomegalovirus antibody, response to prompt reduction of the steroid dose as observed; antiviral therapy was not required. Adverse events associated with this combined therapy have been previously reported in patients with rheumatoid arthritis. The incidence of adverse effects with TAC+ anti‐TNFα therapy was no higher than that demonstrated by postmarketing surveillance of TAC monotherapy.^18^, ^19^

In patients with rheumatoid arthritis, TAC is administered at a set dose, which is not based on the trough level. Thus, the dosage of TAC for combined therapy in our study was different from that for combined therapy in patients with rheumatoid arthritis, in whom the usual dose of TAC is 1–3 mg/day.^19^, ^20^ In contrast, during the initial 2 weeks of treatment for UC, TAC is administered at a dose that maintains a high trough level of 10–15 ng/dL; after this, the dose of TAC is adjusted to achieve a trough level of 5–10 ng/dL.^2^ Accordingly, the dose of TAC varies among UC patients, but doses used for the treatment of UC are far higher than the dose of 3 mg/day used for rheumatoid arthritis. In fact, the mean dose of TAC was 6.1 ± 3.6 mg/day in the present study. Because of this difference in the dose of TAC between our study and reports on patients with rheumatoid arthritis, the safety of combined therapy for UC still needs further careful evaluation.

Side effects of concern in combination therapy include renal impairment, cardiac dysfunction, glucose tolerance impairment, infection, and psychoneurological disorder. Care must be taken with regard to side effects.

In recent years, it has been reported that vedolizumab, an α4β7 integrin monoclonal antibody, is effective for UC, and concomitant administration of vedolizumab with TAC has been studied.^22^ While TAC plus vedolizumab was used as a bridging therapy in such studies, the treatment schedule differed from that of our study. Nonetheless, it is interesting and noteworthy that a combination of TAC and a biologic drug was used as a bridging therapy to induce and maintain remission in UC.

In the future, it will be important to further evaluate the course of UC after remission has been induced by TAC + anti‐TNFα therapy. Regarding the other limitations of this study, it was a retrospective investigation conducted at a single institution. As a result, there might be various biases in our data. Accordingly, a prospective multicenter study should be conducted to evaluate the efficacy and safety of TAC + anti‐TNFα therapy in the future.

In conclusion, TAC + anti‐TNFα therapy was effective in more than half of the patients with refractory UC who had not responded to monotherapy. The relapse rate was 33.3% at 1 year after remission induction, and the combined therapy did not cause any serious complications. Our findings suggest that combination TAC + anti‐TNFα therapy may be an option as a new third treatment for refractory UC. The efficacy and safety of this combination therapy should be confirmed in a new, prospective, multicenter trial.
